# Neoadjuvant docetaxel, oxaliplatin plus capecitabine versus oxaliplatin plus capecitabine for patients with locally advanced gastric adenocarcinoma: long-term results of a phase III randomized controlled trial

**DOI:** 10.1097/JS9.0000000000000692

**Published:** 2023-09-02

**Authors:** Yuan Tian, Peigang Yang, Honghai Guo, Yang Liu, Ze Zhang, Pingan Ding, Tao Zheng, Huiyan Deng, Wenqian Ma, Yong Li, Liqiao Fan, Zhidong Zhang, Dong Wang, Xuefeng Zhao, Bibo Tan, Yu Liu, Qun Zhao

**Affiliations:** a The Third Department of Surgery; b Department of Endoscopy; c Department of Pathology, The Fourth Hospital of Hebei Medical University; d Hebei Key Laboratory of Precision Diagnosis and Comprehensive Treatment of Gastric Cancer, Shijiazhuang, People’s Republic of China

**Keywords:** advanced gastric cancer, capecitabine, docetaxel, oxaliplatin, neoadjuvant chemotherapy, pathological complete response, prognosis

## Abstract

**Background:**

Neoadjuvant chemotherapy with docetaxel, oxaliplatin, and capecitabine (DOX regimen) is rarely used in Eastern countries and its efficacy and safety in advanced gastric cancer have not been reported. In this open-label, randomized, controlled trial, the authors aimed to assess the clinical efficacy of neoadjuvant chemotherapy using the DOX and oxaliplatin plus capecitabine (XELOX) regimens, in comparison to surgery alone.

**Materials and methods:**

Three hundred patients younger than 60 years with potentially resectable advanced gastric cancer (cT3-4, Nany, M0) were enrolled in this randomized controlled clinical trial between November 2014 and June 2018. The primary endpoint of the study was the pathological complete response (pCR) rate. Secondary endpoints included 3-year overall survival (OS), 3-year disease-free survival.

**Results:**

In total, 280 patients (93 in the DOX group, 92 in the XELOX group, and 95 in the surgery group) were included in the per-protocol analysis. The DOX group demonstrated a significantly higher pCR rate compared to the XELOX group (16.1 vs. 4.3%, *P*=0.008). For patients with intestinal type, the DOX group exhibited significantly higher rates of both pCR and major pathological response compared to the XELOX group (*P*=0.007, *P*<0.001). The 3-year OS rates of the DOX group, the XELOX group and the surgery group were 56.9, 44.6, and 34.7%, respectively. The 3-year disease-free survival rates were 45.2, 40.2, and 28.4%, respectively. The neoadjuvant DOX regimen demonstrated a significant improvement in the 3-year OS of patients compared to the neoadjuvant XELOX regimen (*P*=0.037).

**Conclusion:**

The neoadjuvant DOX regimen has shown the potential to increase the pCR rate and improve the prognosis of patients with advanced gastric cancer who are under 60 years old.

## Introduction

Highlights

- The pathological complete response rate of the neoadjuvant DOX regimen was higher than that of the XELOX regimen.
- The 3-year survival of the neoadjuvant DOX regimen was superior to that of the XELOX regimen and the surgery group.
- Neoadjuvant chemotherapy with the DOX regimen is well tolerated in patients with advanced gastric cancer.

Gastric cancer is one of the major malignant tumors that threaten the lives and health of people all over the world. In 2020, according to the WHO, the incidence and mortality of gastric cancer ranked fifth and fourth, respectively. In addition, China reported ~479 000 new cases of gastric cancer, resulting in 374 000 deaths. These numbers account for 44.0% of new cancer cases and 48.6% of cancer-associated deaths worldwide^1^. The majority of patients are diagnosed with advanced gastric cancer, which is associated with a poor prognosis. The standard treatment approach for locally advanced gastric cancer varies worldwide. In Western countries, the preferred options include perioperative chemotherapy or postoperative adjuvant chemoradiotherapy, while in Asian countries, the main treatment involves a combination of perioperative chemotherapy with D2 gastrectomy^2-8^. At present, capecitabine combined with oxaliplatin (XELOX) is a recommended regimen for perioperative chemotherapy in advanced gastric cancer^9^. In our previous study, we found that the S-1 combined with oxaliplatin (SOX) and XELOX regimens exhibited equal levels of activity and were well tolerated^10^. A retrospective analysis revealed that for patients with locally advanced gastric cancer, the DOS (docetaxel, oxaliplatin, and S-1) regimen demonstrated greater benefits than the XELOX regimen as neoadjuvant chemotherapy, with no additional toxicity effects^11^.

In recent years, the combination of oxaliplatin and fluorouracil with docetaxel has been increasingly utilized in neoadjuvant chemotherapy^12^. Furthermore, this regimen is recommended in the guidelines of the National Comprehensive Cancer Network (NCCN) and the European Society for Medical Oncology^13,14^. However, there is currently a lack of extensive clinical data to ascertain whether Chinese patients can exhibit enhanced tolerance to docetaxel-based triplet chemotherapy and achieve significant improvements in prognosis. As a result, we were prompted to conduct a randomized controlled clinical trial (NCT02555358) to assess the combination of oxaliplatin and capecitabine with docetaxel (DOX), comparing perioperative DOX and XELOX with surgery alone. The short-term results of this study were published in 2021^15^.

In this study, we explore the longer-term outcomes, including overall survival (OS), progression-free survival, and subgroup analyses.

## Materials and methods

### Study design and participants

This study was a randomized, open-label controlled trial conducted in accordance with the principles of the Helsinki Declaration. Patients were enrolled between 15 November 2014 and 26 June 2018. Eligible patients were 18–60 years of age, with an Eastern Cooperative Oncology Group performance status of 0–1, and a newly histologically confirmed primary gastric or gastroesophageal junction adenocarcinoma that was locally advanced but amenable to curative resection, specifically clinical TNM staging cT3-4 Nany stage.

The study protocol, which includes the complete eligibility criteria, is provided as SDC (Supplementary Digital Content): Trial protocol, Supplemental Digital Content 1, http://links.lww.com/JS9/A933. This study was presented according to the CONSORT guidelines^16^ (Supplemental Digital Content 2, http://links.lww.com/JS9/A934), (Supplemental Digital Content 3, http://links.lww.com/JS9/A935).

### Randomization and masking

All patients were centrally randomized in a 1:1:1 ratio to receive neoadjuvant DOX, neoadjuvant XELOX, or surgery alone using an interactive web-response system (IWRS).

Assignment was performed in a face-to-face manner after patients met the inclusion criteria and provided informed consent. Patients and caregivers were not blinded to the assigned intervention after the assignment.

### Procedures

Neoadjuvant DOX or XELOX was administered for 4 cycles, followed by an additional four cycles of postoperative XELOX for the DOX or XELOX group.

The surgery group received a total of eight postoperative cycles of XELOX. The DOX treatment regimen comprised intravenous administration of docetaxel at a dose of 60 mg/m^2^ on day 1, intravenous administration of oxaliplatin at a dose of 130 mg/m^2^ on day 1, and oral administration of capecitabine at a dose of 1000 mg/m^2^ p.o. (in two doses of 500 mg/m^2^ per day) from day 1 to 14, every 3-week cycle. XELOX treatment consisted of intravenous administration of oxaliplatin at a dosage of 130 mg/m^2^ on day 1, along with oral administration of capecitabine at a dosage of 1000 mg/m^2^ (500 mg/m^2^ twice a day) from day 1 to 14, every 3-week cycle. If patients developed febrile neutropenia (despite the use of granulocyte colony-stimulating factor), experienced thrombocytopenia leading to bleeding, or encountered any other hematological dose-limiting toxicities, the dosages of docetaxel and oxaliplatin were reduced to 75%. For nonhematological toxicities of grade >2, the dose of all drugs was reduced to 75%. In the case of grade 2 toxicity recurring after the initial dose reduction, the dose was further reduced to 50%. Treatment was continued until intolerable toxicity, disease progression, patient death, withdrawal of consent, or the investigator’s decision.

Prior to surgical treatment, CT or MRI scans and endoscopy were performed to exclude disease progression or distant metastasis. Responses were evaluated by investigators, radiologists, and pathologists in accordance with RECIST 1.1 criteria^17^.

Surgery was scheduled for 4 to 6 weeks after the completion of the last cycle of neoadjuvant chemotherapy. Radical gastrectomy was performed by one of the five experienced surgeons who conducted at least 100 radical gastrectomy procedures per year.

The tumor regression grade (TRG) was quantified in accordance with the NCCN Clinical Practice Guidelines in Oncology (2014.v1). TRG0 [pathological complete response (pCR)] was defined as a complete response without any viable cancer cells. TRG1 was characterized as a near-complete response with single cells or rare small groups of cancer cells. TRG2 indicated a partial response with residual cancer cells showing evident tumor regression but more than single cells or rare small groups of cancer cells. TRG3 represented a poor or no response with extensive residual cancer without evident tumor regression. The major pathological response (MPR) was defined as patients with TRG0 and TRG1.

Pathological staging, which includes the assessment of tumor invasion depth (T), lymph node involvement (N), and resection margin status (RX, R0, or R1), was determined by the local pathologist following the guidelines outlined in the 7th edition of the TNM American Joint Committee on Cancer (AJCC) classification^18^. Adverse events were assessed in accordance with the National Cancer Institute Common Terminology Criteria for Adverse Events (NCI-CTC, version 3.0). Intraoperative incidents were analyzed using the Satava classification^19^, while postoperative complications were classified according to the Clavien–Dindo classification^20^.

The primary endpoint was the pCR rate. Secondary endpoints included the 3-year OS and 3-year disease-free survival (DFS). OS was defined as the time from randomization to death from any cause, while DFS was defined as the time from randomization to disease recurrence.

### Sample size and statistical analysis

Based on the results of previous trials^10,21^, we anticipated a 5% pCR rate in the XELOX group. We expected the DOX regimen to increase the pCR rate to 15% in this group. A sample size of 100 patients per group was calculated to provide 80% power to detect this improvement in pathological complete regression (using a one-sided significance level of *P*<0.05; Fisher’s exact test), accounting for a dropout rate of 15%.

Participants who were randomly assigned but did not receive chemotherapy or surgery were excluded from all analyses. All patients who underwent randomization were defined as the intention-to-treat (ITT) population. The remaining participants were included in the modified intention-to-treat (mITT) population, which included all participants who were randomly assigned and received any form of study treatment. The originally specified analysis population in the protocol was ITT, while efficacy and survival analyses were conducted in the mITT population. Safety analyses were performed on patients who received at least one dose of the assigned treatment.

The *χ* ^2^ test was used to compare classification data between the treatment groups. For continuous variables, the Kolmogorov–Smirnov test was utilized, and comparisons were made using either the unpaired *t*-test or the Mann–Whitney *U* test. Time-to-event curves for disease-free and OS were calculated using the Kaplan–Meier method. Follow-up was censored at 3 years postrandomization. A two-sided *P*<.05 was considered statistically significant. Statistical analyses were conducted using SAS software (version 9.3).

## Results

### Trial design and enrollment

**Figure 1:**
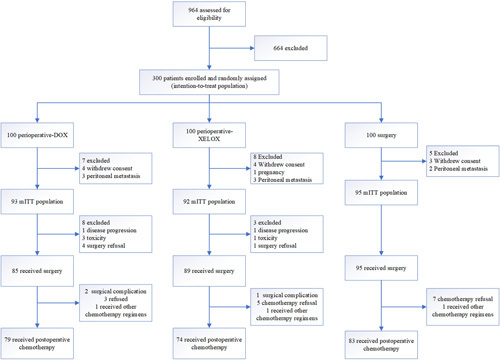
CONSORT diagram. DOX, docetaxel, oxaliplatin, and capecitabine; XELOX, oxaliplatin plus capecitabine.

One hundred patients per group were enrolled between November 2014 and June 2018 (Fig. 1, Table 1). Twenty (6.7%) patients withdrew from the study without receiving any study drug or surgery. As a result, 93, 92, and 95 patients entered the mITT population in the DOX, XELOX, and surgery groups, respectively. Of these, 85, 89, and 95, respectively, underwent surgery. Finally, 79, 74, and 83 patients per group received adjuvant chemotherapy (Fig. 1). The baseline characteristics of the three groups were well balanced (Table 1). The original publication included information on radiological response, adverse effects of chemotherapy, and perioperative complications^8^ (SDC, Table 1, Supplemental Digital Content 4, http://links.lww.com/JS9/A936, 2, Supplemental Digital Content 5, http://links.lww.com/JS9/A937, 3, Supplemental Digital Content 6, http://links.lww.com/JS9/A938, 4, Supplemental Digital Content 7, http://links.lww.com/JS9/A939, 5, Supplemental Digital Content 8, http://links.lww.com/JS9/A940).

**Table 1:** Demographic data in the three groups.

Parameter	DOX (*n*/%) *N*=100	XELOX (*n*/%) *N*=100	SURGERY(*n*/%) *N*=100	*χ* ^2^	*P*
Age (year)				1.520	0.468
<45	15(15)	17(17)	11(11)		
45–60	85(85)	83(83)	89(89)		
Sex				0.694	0.707
M	73(73)	69(69)	74(74)		
F	27(27)	31(31)	26(26)		
ECOG				0.770	0.681
0	38(38)	42(42)	44(44)		
1	62(62)	58(58)	56(56)		
Tumor center				5.768	0.450
EGJ	33(33)	38(38)	28(28)		
Gastric body	11(11)	14(14)	10(10)		
Gastric antrum	52(52)	42(42)	53(53)		
Other	4(4)	6(6)	9(9)		
Pathological type				2.362	0.669
High and medium differentiation	56(56)	49(49)	51(51)		
Poor differentiation	33(33)	42(42)	36(36)		
Very low differentiation	11(11)	9(9)	13(13)		
cT stage (pre-NAC CT)				1.510	0.470
cT3	28(28)	31(31)	36(36)		
cT4	72(72)	69(69)	64(64)		
cN stage (pre-NAC CT)				7.382	0.287
cN0	10(10)	16(16)	12(12)		
cN1	35(35)	32(32)	41(41)		
cN2	41(41)	46(46)	35(35)		
cN3	14(14)	6(6)	12(12)		
Borrmann type				9.769	0.135
I	3(3)	0	0		
II	35(35)	39(39)	31(31)		
III	55(55)	56(56)	66(66)		
IV	7(7)	5(5)	3(3)		

Very low differentiation: Signet ring cell carcinoma, Mucous adenocarcinoma, Anaplastic carcinoma.

Analysis of intention-to-treat (ITT) population.

### Clinicopathological results

After receiving neoadjuvant chemotherapy, 85, 89, and 95 patients in the DOX, XELOX, and surgery groups, respectively, proceeded to undergo surgery. The surgery was performed after a median of 4 weeks (IQR 3–5) since the last chemotherapy cycle in the DOX group and 4 weeks (2–5) in the XELOX group. Finally, radical resection was performed on 83, 85, and 90 patients in each respective group. While the T stage and N stage of baseline characteristics were similarly distributed across the three groups, the DOX group exhibited a higher proportion of stage ypT0 and pCR compared to the XELOX group [15 (16.1%) of 93 patients vs. 4 (4.3%) of 92 patients; *P*=0.008]. In the XELOX group, all four patients with pCR exhibited tumor-free lymph nodes (ypN0). However, in the DOX group, 2 of 15 patients with pCR, there were histopathological indications of lymph node involvement (ypN1), which were classified as nodal partial regression (nodal TRG 2). We have associated the tumor regression grade with Lauren’s classification.

**Figure 2:**
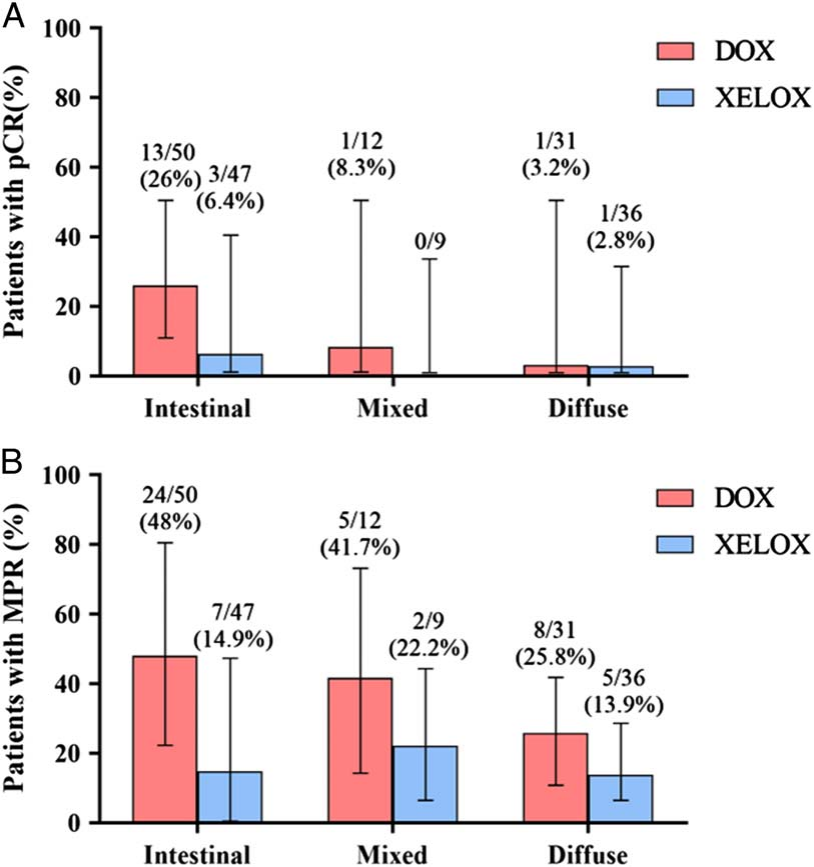
Histopathological regression by Lauren’s classification (A) Pathological complete response (pCR) and (B) major pathological response (MPR) by treatment group according to Lauren’s classification subtype. DOX, docetaxel, oxaliplatin, capecitabine; XELOX, oxaliplatin, capecitabine.

In the pooled mITT population of the DOX and XELOX group, pCR was most commonly observed in intestinal type tumors [16 (16.5%) of 97 patients], while it was least frequent in diffuse type tumors [2 (2.9%) of 67 patients; *P*=0.007]. Additionally, 1 (4.7%) of 21 patient with mixed type histology achieved pCR (*P*=0.17). In the DOX group, 13 (26%) of 50 patients achieved pCR in the intestinal type, while in the XELOX group, 3 (6.4%) of 47 patients exhibited pCR in the intestinal type (*P*=0.009; Fig. 2). Moreover, in the DOX group, 1 (3.2%) of 31 patients had pCR in the diffuse type, whereas in the XELOX group, 1 (2.8%) of 36 patients demonstrated pCR in the diffuse type (*P*=0.91). In the DOX group, 1 (8.3%) of 12 patients had a pCR in the mixed type, while none of the patients in the XELOX group achieved a pCR in the mixed type (*P*=0.38).

The MPR rate for patients with intestinal type was significantly higher in the DOX group than in the XELOX group (48 vs. 14.9%, *P*<0.001), whereas the MPR rate for patients with diffuse and mixed types was similar between the two groups (diffuse: *P*=0.22, mixed: *P*=0.35). The R0 resection rate and pathological N stage did not exhibit any significant differences among the three groups (*P*>0.05) (Table 2).

**Table 2:** Clinicopathological results in three groups.

Parameter	DOX (*n*/%) *N*=93	XELOX (*n*/%) *N*=92	SURGERY (*n*/%) *N*=95	*P*
Proceeded to laparoscopic exploration after NAT	85(91.4)	89(96.7)	95(100)	
Achieved margin-free (R0) resection	83(89.2)	85(92.4)	90(94.7)	0.374
*P*/yp T stage				<0.001
T0	15(16.1)	4(4.3)	0	
T1	9(9.7)	11(11.9)	6(6.3)	
T2	14(15.1)	10(10.9)	7(7.4)	
T3	6(6.5)	8(8.7)	12(12.6)	
T4	39(41.9)	54(58.7)	70(73.7)	
Not applicable^a^	10(10.8)	5(5.4)	0	
*P*/yp N stage				0.516
N0	31(33.3)	25(27.2)	21(22.1)	
N1	21(22.6)	27(29.3)	30(31.6)	
N2	17(18.3)	19(20.7)	25(26.3)	
N3	14(15.1)	16(17.4)	19(20)	
Not applicable^a^	10(10.8)	5(5.4)	0	
Pathological regression grade				<0.001
TRG0	15(16.1)	4(4.3)		
TRG1	24(25.8)	17(18.5)		
TRG2	26(28)	36(39.1)		
TRG3	28(30.1)	35(38.1)		

Analysis of mITT population except otherwise indicated.

a Includes patients who could not be staged due to no operation, palliative surgery, or others.

### Follow-up

**Figure 3:**
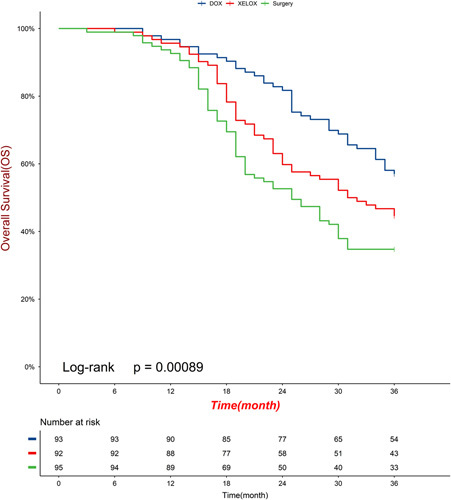
The 3-year overall survival analysis in mITT population (A) DOX versus XELOX, (B) XELOX versus surgery. DOX, docetaxel, oxaliplatin, capecitabine; XELOX, oxaliplatin, capecitabine.

**Figure 4:**
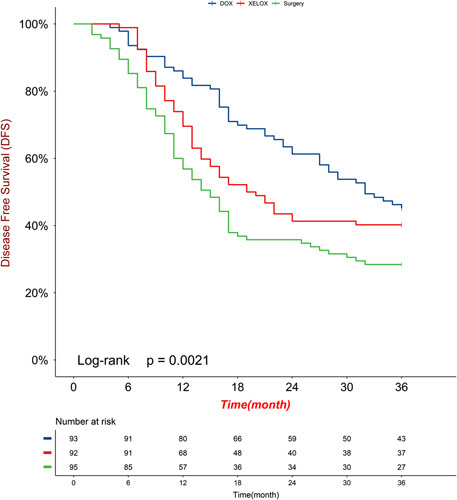
The 3-year disease-free survival analysis in mITT population (A) DOX versus XELOX, (B) XELOX versus surgery. DOX, docetaxel, oxaliplatin, capecitabine; XELOX, oxaliplatin, capecitabine.

**Figure 5:**
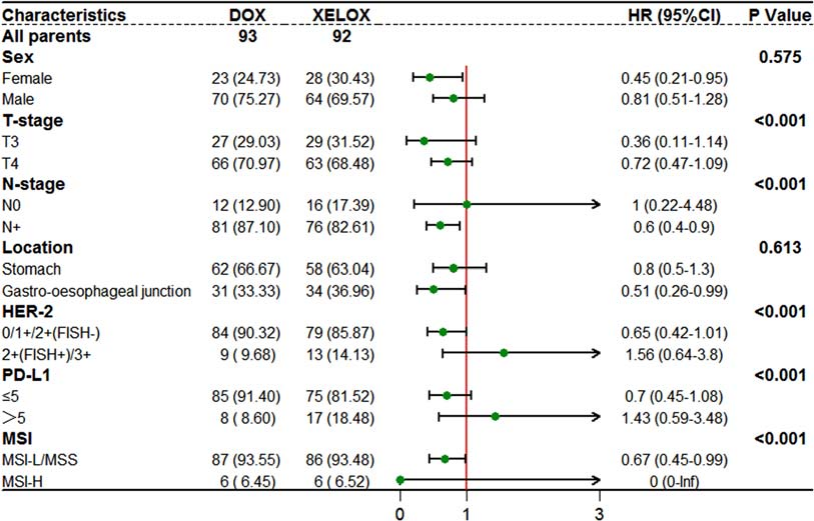
Subgroup analyses of disease-free survival in DOX and XELOX. DOX, docetaxel, oxaliplatin, capecitabine; XELOX, oxaliplatin, capecitabine.

**Figure 6:**
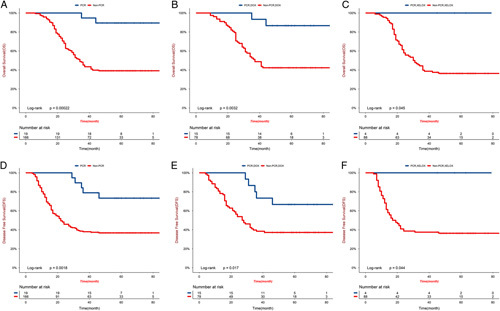
Kaplan–Meier estimates of overall survival (A,B,C) and disease-free survival (D,E,F) according to the tumor regression grade (A) Overall survival in the pooled population (DOX and XELOX group) (B) Overall survival in the DOX group (C) Overall survival in the XELOX group (D) Disease-free survival in the pooled population (DOX and XELOX group) (E) Disease-free survival in the DOX group (F) Disease-free survival in the XELOX group. pCR, pathological complete response; Non-pCR, nonpathological complete response; DOX, docetaxel, oxaliplatin, capecitabine; XELOX, oxaliplatin, capecitabine.

The median follow-up for surviving patients was 70.8 months (range 48.3–92.7; IQR 59.4–81.6), with 14 (5%) of 208 patients lost to follow-up (five, three, and six patients in the DOX, XELOX, and surgery groups, respectively). During the follow-up period, 44, 36, and 28 patients per group were still alive. In the DOX group, 49 patients died (44 deaths due to gastric cancer and 5 deaths due to other reasons). In the XELOX group, 56 patients died (52 deaths due to gastric cancer, 4 deaths due to other reasons). In the surgery group, 67 patients died (63 deaths due to gastric cancer, 3 deaths due to other reasons, and 1 death occurring within 30 days of surgery). The 3-year OS rates of the DOX group, the XELOX group, and the surgery group were 56.9, 44.6, and 34.7%, respectively. The 3-year disease-free survival rates were 45.2, 40.2, and 28.4%, respectively. The hazard ratio (HR) comparing OS between the DOX group and the XELOX group was 0.64 (95% CI: 0.42–0.97; *P*=0.037; Fig. 3). The HR comparing OS between the XELOX group and the surgery group was 0.72 (95% CI: 0.49–1.05; *P*=0·091; Fig. 3). The HR comparing DFS between the DOX group and the XELOX group was 0.75 (95% CI: 0.50–1.10; *P*=0.139; Fig. 4). The HR comparing DFS between the XELOX group and the surgery group was 0.69 (95% CI: 0.48–0.99; *P*=0.049; Fig. 4). Figure 5 shows the subgroup analyses of OS between the DOX group and the XELOX group. Our survival analysis revealed that patients who achieved a pCR had significantly longer OS time and disease-free time compared to non-pCR patients. This difference was found to be statistically significant (all *P*<0.05), as illustrated in Figure 6.

## Discussion

Docetaxel-based neoadjuvant chemotherapy regimens for gastric cancer have been extensively studied in clinical trials, with most of the evidence derived from Western countries^12,22^. In China, there have been limited clinical trials exploring docetaxel-based triplet neoadjuvant chemotherapy, and only a retrospective analysis demonstrated that the FLOT regimen exhibited a better survival improvement^23^. A prospective clinical study conducted in China reported no statistically significant difference in pathological regression grade between the docetaxel-based FLOT and SOX regimens. It is important to note that this study had a small sample size and lacked long-term follow-up results^24^. While these findings suggest that docetaxel-based triplet chemotherapy holds promise as a neoadjuvant regimen, caution is required to ensure its tolerability in Asian patients, who are more susceptible to docetaxel-induced myelosuppression compared to their White counterparts^25^. Therefore, our study established the minimum inclusion criteria, enrolling patients under 60-year-old with an ECOG score of 0–1.

Based on the findings of our previous clinical study comparing neoadjuvant chemotherapy with XELOX versus SOX, we found that the XELOX regimen had satisfactory safety, tolerability, and a certain level of clinical efficacy^10^. Based on these results, our aim was to achieve improved clinical efficacy while ensuring patients can tolerate the treatment adequately. Therefore, in this study, we have chosen the DOX regimen.

The proportion of pathological regression grade, specifically the pCR rate in the XELOX group, was consistent with the results of the previous trial^10^. Limited prospective clinical studies have focused on neoadjuvant chemotherapy with the DOX regimen. However, in our study, we observed that the pCR rate in the DOX group was comparable to the results reported in the FLOT4 trial for the FLOT regimen^26^. Moreover, the pCR rate in the DOX group was slightly higher than the pCR rate achieved with the docetaxel, oxaliplatin, and S-1 neoadjuvant regimen in the PRODIGY trial^27^. Notably, this result is consistent with the findings of our prior clinical study on neoadjuvant concurrent chemoradiotherapy for potentially resectable advanced Siewert type II and III adenocarcinomas of the esophagogastric junction^28^.

We observed a significant difference in histopathological regression between diffuse and intestinal type tumors. In the pooled population analysis, intestinal tumors were found to be more likely to achieve a pCR. These results were consistent with those of a previous study^29^. DOX demonstrated the highest efficacy compared to XELOX for patients with intestinal type tumors. Additionally, DOX exhibited a higher rate of MPR in patients with intestinal tumors. These findings suggest that DOX may contribute to the identification of precise individualized treatment strategies. However, the determination of Lauren’s classification may not be definitive in all patients at the time of gastric cancer diagnosis, and the availability of fewer tissue samples can potentially impact the accuracy of the diagnosis. In order to enhance the diagnostic efficacy, endoscopists were provided instructions to obtain as many biopsies as possible to confirm Lauren’s classification.

pCR, as a short-term indicator for predicting the efficacy of neoadjuvant chemotherapy, enables faster conclusions to be drawn and facilitates decisions on whether further research should be pursued. Moreover, pCR is less susceptible to selection bias and is minimally influenced by the quality of surgery. However, the question of whether pCR can replace long-term survival as a predictor of efficacy remains a topic of ongoing controversy^30,31^.

In this study, the pCR rate in the DOX group was significantly higher than that in the XELOX group, and the 3-year OS rate was also significantly improved compared to XELOX. These findings demonstrate the consistency between pCR and OS. Stratified analysis of the pCR and non-pCR populations revealed that patients with pCR had significantly longer OS and DFS in both the pooled population and within each group. Therefore, pCR is indeed considered a crucial indicator for predicting the efficacy of neoadjuvant chemotherapy, and it may even have the potential to replace OS.

One reason for the survival advantage of DOX over XELOX is the role of docetaxel in the treatment of advanced gastric cancer. The key mechanism of docetaxel involves increasing the polymerization of tubulin into stable microtubules while simultaneously reducing depolymerization, without altering the number of protofilaments in the microtubule during these processes. The activity of docetaxel in this disease was established in the early 1990s. Docetaxel monotherapy has shown a response rate of 18–24% as a first-line treatment. Additionally, the combination of docetaxel with cisplatin or oxaliplatin and a fluorouracil chemotherapy regimen has demonstrated effectiveness in the treatment of advanced gastric cancer^11,12,15,21-24,26,27,29^.

In the subgroup analysis of the DOX group and the XELOX group, we observed a survival benefit associated with the DOX regimen in the majority of subgroups. However, patients with HER2-positive and PD-L1 high expression did not achieve significant survival improvement after neoadjuvant chemotherapy with the DOX regimen. This suggests that chemotherapy alone may be difficult to improve the prognosis for these patients, and the addition of targeted therapy or immunotherapy may be necessary. This also represents the research direction for future large randomized controlled clinical studies. These results provide a theoretical basis for precise and individualized treatment of gastric cancer.

This study has some limitations. Based on the survival analysis, neoadjuvant XELOX did not exhibit a superior survival benefit compared to surgery alone; however, a significant benefit in terms of DFS was observed. This may be attributed to the unavailability of 5-year survival data in this study. Therefore, further follow-up is necessary to determine the efficacy of neoadjuvant chemotherapy with more reliable evidence.

In conclusion, based on our analysis, neoadjuvant DOX significantly improved 3-year OS compared to neoadjuvant XELOX and surgery alone in patients with advanced gastric cancer who are younger than 60 years old and have good general fitness. This finding suggests that neoadjuvant DOX has the potential to become a standard treatment approach for this patient population.

## Ethical approval

The present study was approved by the Ethical Review Committee of Hebei Medical University (Shijiazhuang, China) (No.2014020).

## Consent

Written informed consent was obtained from the patient for publication and any accompanying images.

## Sources of funding

This study was funded by the Cultivating Outstanding Talents Project of Hebei Provincial Government Fund (No.2019012); Hebei public health committee county-level public hospitals suitable health technology promotion and storage project (No.2019024); Hebei Medical University Education and Teaching Research Project (No.2020CGPY-12, No.2020CHYB-23); Hebei University Science and Technology Research Project (No.ZD2019139).

## Author contribution

Q.Z. and Y.T.: designed the study; Y.T.: collected the patient data, and drafted the paper; P.G.Y., H.H.G., Y.L., Z.Z., P.A.D., and T.Z.: participated in the design of the study and edited the final paper; Y.T., H.Y.D., W.Q.M., Y.L. (Yang Liu), L.Q.F., Z.D.Z., D.W., X.F.Z., B.B.T., and Y.L. (Yu Liu): analyzed and interpreted the data; Q.Z.: contributed critical revision of the manuscript for important intellectual content and supervised this study. All authors read and approved the paper for publication.

## Conflicts of interest disclosure

The authors declare that they have no conflicts of interest.

## Research registration unique identifying number (UIN)

1. Name of the registry: Three Drugs in Advanced Gastric Cancer Neoadjuvant Chemotherapy for Stage Ⅲ Multicenter, Open, Randomized, Controlled Clinical Study.
2. Unique identifying number or registration: ID:NCT02555358.
3. Hyperlink to your specific registration (must be publicly accessible and will be checked): https://clinicaltrials.gov/ct2/show/NCT02555358?cond=NCT02555358&draw=2&rank=1

## Guarantor

Qun Zhao.

## Data availability statement

The datasets used and/or analyzed during the current study are available from the corresponding author on reasonable request.

## Provenance and peer review

Not commissioned, externally peer-reviewed.

## Supplementary Material

SUPPLEMENTARY MATERIAL
