# Supplementary material for: Neoadjuvant docetaxel, oxaliplatin plus capecitabine versus oxaliplatin plus capecitabine for patients with locally advanced gastric adenocarcinoma: long-term results of a phase III randomized controlled trial
Source: Int J Surg. 2023 Sep 2;109(12):4000–8. doi: 10.1097/JS9.0000000000000692 (PMC10720837; doi:10.1097/JS9.0000000000000692)
Supplement: SUPPLEMENTARY MATERIAL [file js9-109-4000-s001.docx]

**Trial protocol**

**Drug Substances:** Docetaxel, Oxaliplatin, Capecitabine

**Three Drugs in Advanced Gastric Cancer Neoadjuvant Chemotherapy for Stage Ⅲ Multicenter, Open, Randomized, Controlled Clinical Study**

**Study No.: Alien Craft 0004**

**Version No.: v1.1**

**Date: 20 Jun 2014**

| **STATEMENT OF CONFIDENTIALITY**  The information contained in this document is owned by the Sponsor and is now secretly provided to you, your colleagues (including the Ethics Committee) and regulatory authorities. Without the prior written approval of the Sponsor, the content of this document shall not be disclosed to any third party, unless an informed consent is obtained from the subject or it is required by other necessary circumstances. |
| --- |

**TABLE OF CONTENTS**

1. Project summary 4

- 1. Epidemiology4
  2. postoperative adjuvant treatment4
  3. neoadjuvant chemotherapy4

1.4 Background5

2. Study goals and objectives 5

2.1 Primary Outcome Measure5

2.2 Secondary Outcome Measures6

2.3 Study design6

2.4 Eligibility6

3. Criteria6

3.1 Inclusion Criteria6

3.2 Exclusion Criteria7

4. Assessment during the Study Treatment Period7

5. Study Treatment Period8,9

6. Chemotherapy regimens10

6.1 Preparation and administration10

6.2 Treatment of the hypersensitivity reaction to Docetaxel11

6.3 Dose adjustment12,13,14,15

7. Tumor assessment15

8. Surgery15

8.1 Pre-operative assessment15

8.2 Surgery16

8.3 Post-operative assessment16

9. Research procedures and data collection16

9.1 Data collection plan16

9.2 Screening visits17

9.3 Visit during treatment17

9.4 Follow-up visits17

9.5 Follow-up of adverse events18

10. Safety Evaluation18

10.1 Observation of adverse events18

10.2 AE classification18

10.3 AE18

10.4 Judgment of the relationship between AE and experimental drugs18

10.5 SAE18

10.5.1 Definition of serious adverse events18

10.5.2 pregnancy19

10.5.3 Disease progression19

10.5.4 Other anti-tumor treatments19

10.5.5 Hospitalization19

10.5.6 Drug overdose20

10.5.7 SAE reporting procedures20

11 Statistical Analysis20

11.1 Statistical analysis of data sets20

- 1. Statistical analysis plan21

12. Quality control and quality assurance21

1. **Project summary**

1.1 The current situation regarding the incidence of gastric cancer does not portray an optimistic outlook, with a grim prognosis for advanced cases. Surgical resection alone falls short of resolving practical issues.

Gastric cancer is a prevalent digestive tract tumor, ranking third among malignant tumors in developed countries and second in China. Every year, there are approximately 200,000 new cases reported in China. Presently, the etiology of gastric cancer remains unclear. However, its significant heterogeneity, as well as the involvement of multiple factors and steps in its carcinogenesis, contribute to the poor prognosis and limited therapeutic efficacy. This is particularly true for advanced gastric cancer.

After undergoing surgery alone, the 5-year survival rate for early gastric cancer can range from 85% to 90%. However, in China, a significant portion of patients with advanced gastric cancer, up to 70%, are unable to undergo radical resection. Even among those who receive radical surgery, 50% to 60% experience recurrence within 2 years after resection. As a result, the overall 5-year survival rate remains low, ranging from 10% to 30%. The prognosis for advanced gastric carcinoma is dire, making it a focus of current research efforts. There is an ongoing pursuit and exploration of new treatment approaches for advanced gastric cancer, which has emerged as a prominent research area in the field of oncology.

1.2 The notable advantages of adjuvant chemotherapy, in comparison to simple surgery, have solidified the role of drug-assisted therapy in the comprehensive treatment of advanced gastric cancer. This development has also initiated the era of perioperative treatment. Currently, adjuvant chemotherapy has become the standard option following D2 surgery for advanced gastric cancer.

In 2009, the Japanese ACTS-GC study confirmed that patients with stage II and III gastric cancer who underwent D2 surgery and received adjuvant chemotherapy had a higher 3-year survival rate (80.5% vs. 70.1%, P = 0.0024) and a lower risk of death. Despite being a single drug, it preliminarily demonstrates the value of postoperative adjuvant therapy for advanced gastric cancer and sets a precedent for its use. The study also compared the results with CLASSIC, suggesting that capecitabine in combination with oxaliplatin is suitable for stage III patients, with a shorter treatment period and better tolerability. These findings have been incorporated into the latest "Guidelines for Clinical Practice of NCCN Gastric Cancer" (2012 edition) as the primary basis for recommending post-D2 radical gastrectomy treatment plans. In China, the Ministry of Health's "Guidelines for the Diagnosis and Treatment of Gastric Cancer" (2011 edition) acknowledge that the capecitabine and oxaliplatin combination adjuvant chemotherapy regimen is more suitable for the treatment needs of Chinese patients. Consequently, the use of combined adjuvant chemotherapy after D2 surgery for advanced gastric cancer has been firmly established and has become routine practice.

1.3 Based on the current study results, neoadjuvant chemotherapy has shown to improve the R0 resection rate and has significant implications for extending survival in certain patients with advanced gastric cancer. Selecting different patient groups with advanced gastric cancer and exploring individualized neoadjuvant therapy has become one of the important research directions for this highly heterogeneous disease.

Many trials have demonstrated that 40% to 75% of operable patients exhibit subclinical metastasis or tumor invasion of adjacent tissues and organs, as determined through clinical examination or surgery. The concept of neoadjuvant chemotherapy was introduced by American scholar Frei in 1982, and in 1989, Wilke et al. pioneered its clinical implementation for gastric cancer, yielding positive outcomes and initiating the era of neoadjuvant chemotherapy for gastric cancer. Subsequent clinical trials of neoadjuvant chemotherapy for gastric cancer have accumulated valuable experiences. To continuously pursue improved efficacy, various aspects of neoadjuvant chemotherapy regimens are being explored and studied. In 2005, the MAGIC study conducted by the American Society of Clinical Oncology (ASCO) led to the incorporation of the ECF regimen as category 1 evidence for neoadjuvant chemotherapy in the NCCN guidelines. Subsequently, numerous studies investigating new regimens in neoadjuvant chemotherapy were conducted, most of which yielded positive results. These studies have demonstrated higher R0 resection rates and improved survival benefits compared to adjuvant therapy alone. However, for diseases with high heterogeneity, single-agent therapy and standardized regimens may fail to address the diverse needs of different populations. Therefore, there is an urgent need to study and explore individualized treatment schemes for distinct patient populations.

1.4 The R0 resection rate alone may not be sufficient to meet the requirements of patients with the potential for a good prognosis, aiming to achieve a significant improvement in survival.

The pathological complete response rate is emerging as an alternative long-term efficacy endpoint for neoadjuvant therapy. Currently, for these patients, a three-drug regimen with high-intensity preoperative chemotherapy may be a more favorable option.

In the previous neoadjuvant therapy studies, R0 resection was one of the main observation targets. However, with the popularization and application of neoadjuvant chemotherapy in clinical practice, we found that R0 resection was no longer an unattainable goal. However, even after obtaining R0 resection, a significant proportion of patients still did not gain greater survival benefits. It was further found that compared with R0 resection, pathological remission rate seemed to have greater significance in improving the prognosis of advanced gastric cancer. There are clear and similar results in breast cancer. Therefore, it is obvious that the current recognized and commonly used neoadjuvant therapy can not meet this requirement, let alone be extended to all patients. For some young patients with advanced gastric cancer who are in good health and can receive high intensity chemotherapy, drugs and regimens with higher pCR rate and RR rate may be a better choice.

**2. Study goals and objectives**

2.1 Primary Outcome Measure:

2.1.1 The pathological complete response rate

[Time Frame: 24 weeks]

2.2 Secondary Outcome Measures:

2.2.1 Overall survival (OS)

[Time Frame: 3 years]

2.2.2 Disease-free survival (DFS)

[Time Frame: 3 years]

2.2.3 Adverse events

The investigators graded all adverse events and toxic effects according to the National Cancer Institute's Common Toxicity Criteria, version 2.0. The number of participants with adverse events will be recorded at each treatment visit.

[Time Frame: 3 years]

2.3 Study design

The sample size calculation for the study was based on the assumption that the proportion of patients achieving pathological complete regression would be 5% with XELOX and 15% with DOX. A total of 300 patients were calculated to provide 80% power to detect this improvement in pathological complete regression (using a one-sided significance level of p<0.05 and Fisher's exact test), accounting for a 15% dropout rate.

2.4 Eligibility

Minimum Age: 20 Years

Maximum Age: 60 Years

Sex: All

Accepts Healthy Volunteers: No

**3. Criteria**

3.1 Inclusion Criteria:

1. Histologically or cytologically proven in operable advanced gastric adenocarcinoma;
2. Subjects who were identified as potentially resectable cases by a multidisciplinary consultation;
3. KPS> 80; ECOG score: 0-1;
4. Expected survival> 6 months;
5. Age 20 -60;
6. Major organ function has to meet the following criteria:
7. Neutrophil count ≥1.5 × 109 / L, platelet count ≥100 × 109 / L, Hemoglobin ≥90g / L, liver function <1.5 times the upper limit of normal, serum bilirubin ≤1.0 × UNL, serum creatinine <1.5 × UNL, PT-INR / PTT <1.7 times the upper limit of normal;
8. Subjects have to voluntarily join the study and sign the Informed Consent Form for the study;

3.2 Exclusion Criteria:

1. Associated with serious diseases in liver, kidney, cardiovascular system and other vital organs;
2. History of hypersensitivity to docetaxel, capecitabine, oxaliplatin or the ingredients of this product;
3. Receiving any form of chemotherapy or other study medication;
4. Pregnancy or lactation women, or women with suspected pregnancy or men unless using a reliable and appropriate contraceptive method;
5. Associated with the inability to swallow, hemorrhagic peptic ulcer, mechanical or paralytic ileus, gastrointestinal active bleeding.

**4. Assessment during the Study Treatment Period**

During the study treatment period, AEs will be continuously monitored until 28 days after the last dose of the study drug.

During the study treatment period, the patients' concomitant diseases, treatments, and compliance with study medication will be continuously monitored.

Each patient will have vital signs, body weight measured, Karnofsky scoring, hematology (including white blood cells/neutrophils), and serum biochemistry tests performed at specified times.

If clinically indicated, ECG and chest X-ray/CT examinations will be performed. During the study treatment period and within 28 days after the last dose of the study drug, all relevant information regarding hospitalization profiles, treatment of AEs, outpatient treatments, and profiles should be documented.

At the end of the study treatment, tumor evaluation (clinical tests, chest X-ray or chest CT/MRI, and abdomen CT/MRI) should be performed. If the patient shows any signs of relapse (e.g., related clinical manifestations), additional tumor evaluation should be conducted during the treatment period. Possible re-surgery and/or further cancer treatment should also be documented.

If the patient is postoperatively confirmed as metastatic GC (e.g., ascitic fluid exfoliative cytology detects tumor cells) or if the patient fails to receive R0 resection, detailed documentation of intraoperative findings, surgical method, and postoperative pathology should be performed regardless of the adopted surgical approach.

All patients should be pathologically staged according to AJCC TNM 7.0. For patients classified as pT1/T2N0M0 or pT1N1M0 (i.e., stage I patients) in arms A and B, postoperative chemotherapy should still be administered according to the study protocol based on a postoperative pathological stage of ypT1/T2N0M0 or ypT1N1M0. Patients in arm C who do not meet the criteria for adjuvant chemotherapy should not receive adjuvant chemotherapy, but tumor evaluation should still be performed according to the schedule to record DFS and OS.

For patients in arms A and B who fail to undergo radical resection of GC (D2 lymph node dissection) due to tumor progression, switching to Paclitaxel-based systemic chemotherapy or receiving treatment based on the investigator's clinical practice should be considered, with DFS and OS recorded. Patients who fail to undergo radical resection of GC (D2 lymph node dissection) for other reasons should continue with SOX chemotherapy and follow the clinical practice of the study site, but DFS and OS should still be recorded.

All patients should initiate chemotherapy within 8 weeks post-op, and if this is not possible, the investigator should select a combination chemotherapy regimen, single-agent chemotherapy, or observation for the arm based on clinical practice. Follow-up should be carried out according to the study protocol, and DFS and OS should be recorded.

**5. Study Treatment Period**


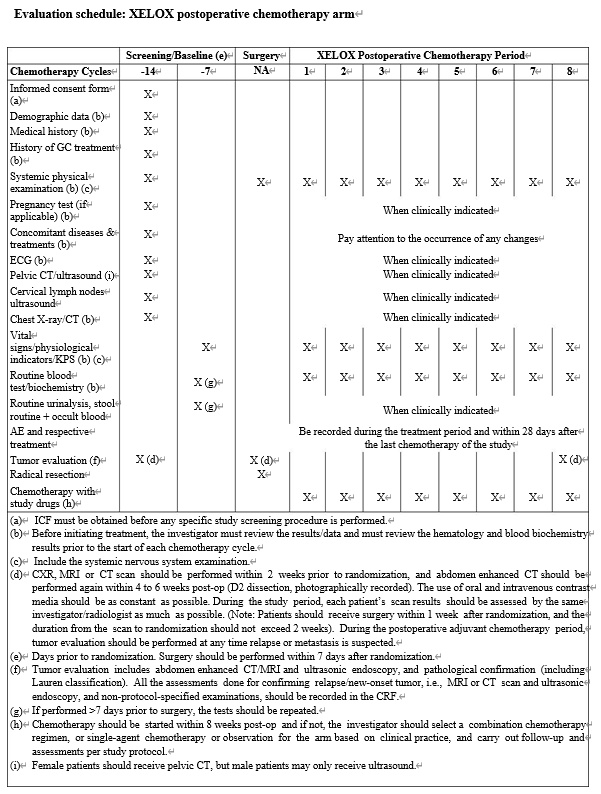


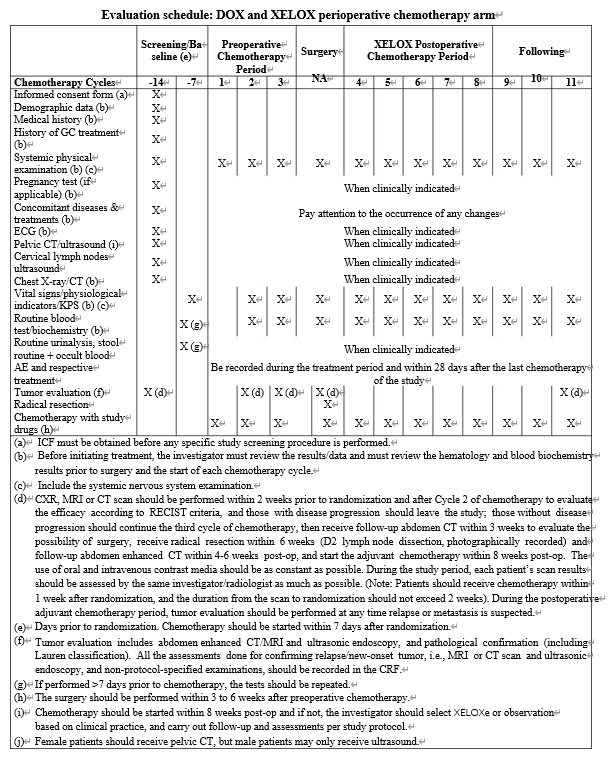


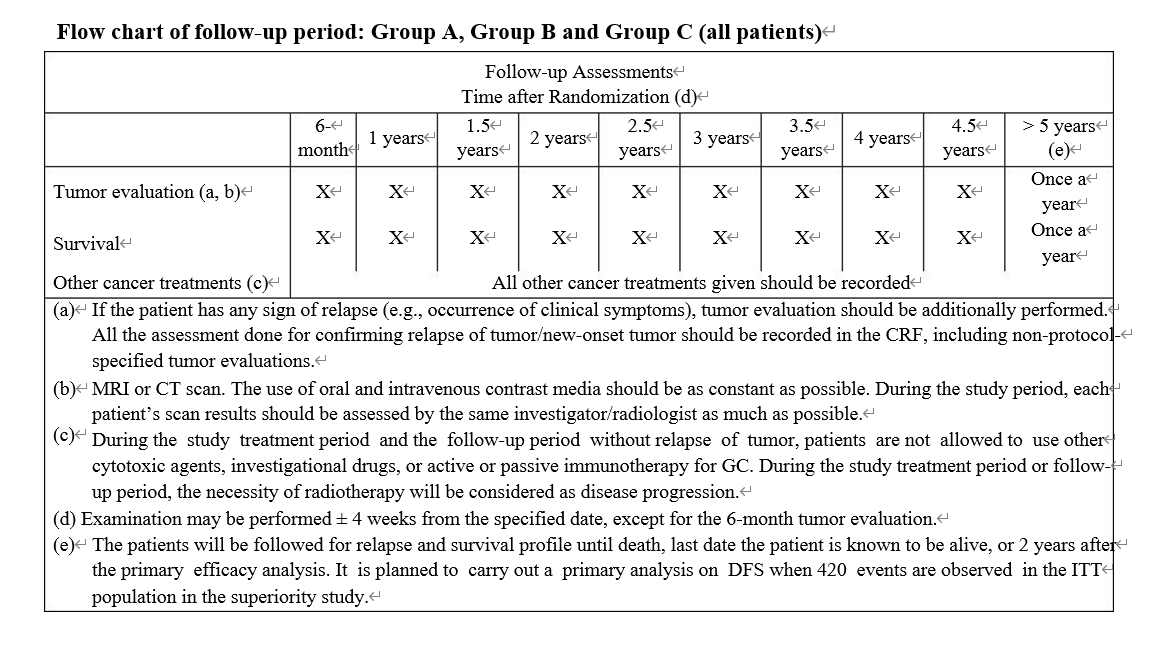


**6. Chemotherapy regimens**

6.1 Preparation and administration

For Docetaxel, Oxaliplatin, and capecitabine, the Mosteller method is used for the BSA formula as follows: (height (cm) is rounded off to an integer, and body weight (kg) is rounded to the first decimal point) BSA (m²) = ([Height (cm) x Weight (kg)] / 3600) ½

The dose is determined based on the BSA measured at the start of each chemotherapy Cycle 1. However, if the BSA on Day 1 of each treatment cycle differs from the BSA at the initiation of Cycle 1 by ≥+/-10%, the re-measured BSA will be used.

Docetaxel: Two types of Docetaxel will be supplied to sites. One is a package (2 vials) in which the Docetaxel study drug and solvent are separately provided to be diluted and mixed at the site, and the other (1 vial) is a form that is previously diluted and mixed at the factory.

The package consisting of the study drug and solvent should be provided to subjects after the following process: If the vials were refrigerated, leave the required Taxotere® package at room temperature for 5 minutes. Hold the vial at an angle, take the entire contents in the solvent vial with a syringe and a needle using an aseptic technique, and infuse the solvent into the Taxotere® vial.

Carefully invert this stock solution upside down for at least 45 seconds and allow it to stand at ambient temperature for 5 minutes. This stock solution contains Docetaxel 10 mg/mL and should be used immediately for the preparation of the infusion fluid.

Several stock solution vials may be required to obtain the appropriate dose (mg) for the subject.

This stock solution is infused into a 200 mL injection bag or an injection bottle containing 5% dextrose or 0.9% sodium chloride.

Docetaxel 50mg/m² is intravenously administered on Day 1 of each 3-week cycle over ≥1 hour.

Rounding off of the calculated dose is determined at the discretion of the investigator at the site.

Pretreatment with Dexamethasone (8 mg) should be concurrently administered to prevent the hypersensitivity reaction and pulmonary/peripheral edema and to reduce and/or delay Docetaxel-related skin toxicity and fluid retention, as follows. Hypersensitivity reaction may develop irrespective of pretreatment.

6.2 Treatment of the hypersensitivity reaction to Docetaxel:

When infusing Docetaxel, it is necessary to use the instillation (dropping) method for the first 5 minutes. The administrator should stay beside the patient's bed and, during the first dose, monitor the patient's general condition while checking the blood pressure and pulse, if possible.

In case of an adverse event, immediate emergency resuscitation should be performed.

As part of the emergency resuscitation procedure, antihistamines, corticosteroids, aminophylline, and epinephrine should be readily available beside the patient.

If a hypersensitivity reaction occurs during Docetaxel administration, appropriate emergency treatment should be initiated based on the symptoms. For the severity and corresponding treatment of the hypersensitivity reaction, refer to the following table.

| Mild symptom: Local adverse events of pruritus, flushing, and rash | Reduce the infusion rate until the patient's condition has recovered.  Stay available next to the patient.  If the patient's condition recovers, resume infusion at the initially planned rate.  For subsequent cycles, infuse at the planned pretreatment and infusion rates. |
| --- | --- |
| Moderate symptom:  Abnormal symptom not  listed in mild or severe  symptoms, generalized  pruritus, flushing, rash,  dyspnea, hypotension  with systolic blood pressure >80 mm Hg | Discontinue the infusion of Docetaxel.  After intravenous infusion of Dexamethasone 10 mg, administer an intravenous infusion of Diphenhydramine 50 mg.  Administer Docetaxel infusion again after the recovery of symptoms.  For subsequent cycles, administer an intravenous infusion of Dexamethasone 10 mg and an intravenous infusion of Diphenhydramine 50 mg as planned pretreatment, and then infuse Docetaxel 1 hour later. |
| Severe symptom:  Bronchospasm,  generalized urticaria, low  blood pressure (systolic  blood pressure  <80mmHg), angioedema  (periocular edema) | Discontinue the infusion of Docetaxel.  Administer an intravenous infusion of Dexamethasone 10 mg, followed by an intravenous infusion of Diphenhydramine 50 mg. Infuse Epinephrine as necessary.  After symptom recovery, infuse Docetaxel within 3 hours. Alternatively, within 72 hours, administer an intravenous infusion of Dexamethasone 10 mg, an intravenous infusion of Diphenhydramine 50 mg, and then infuse Docetaxel 1 hour later.  For subsequent cycles, (1) in the evening of the Docetaxel infusion day, administer Dexamethasone 20 mg orally, (2) in the morning of the Docetaxel infusion day, administer Dexamethasone 20 mg orally 1 hour before, (3) in the morning of the Docetaxel infusion day, administer an intravenous infusion of Diphenhydramine 50 mg 1 hour before, and then infuse Docetaxel. In case of recurrence of severe symptoms, the patient will be excluded from treatment. |
| Anaphylaxis (NCI  CTCAE Grade 4) | Discontinue treatment. |

Note: Docetaxel is an anticancer agent, and like other potentially toxic chemicals, attention should be paid to the handling and preparation of the Docetaxel solution. The investigator should wear gloves, and in case of skin contact with the concentrated stock solution or injection solution of Docetaxel, they should immediately wash thoroughly with soap and running water.

**Oxaliplatin**

Oxaliplatin is reconstituted with water for injection or a 5% dextrose solution, either 10 mL (containing 50 mg of solid substance) or 20 mL (containing 100 mg of solid substance), to obtain a concentration of 5 mg/mL.

The prepared solution is then diluted with the final infusion solution, which can be 200 mL, 250 mL, or 500 mL of 5% dextrose fluid.

Oxaliplatin, administered intravenously, is given at a dose of 100 mg/m^2^ over a period of at least 2 hours on Day 1 of every 3-week cycle. The rounding off of the calculated dose is determined at the discretion of the investigator at the site.

In the case of extravasation, administration should be immediately discontinued, and after removing the needle, the injection site should be kept higher than the heart to minimize tissue damage.

If acute throat paresthesias occur during or after a 2-hour instillation of Oxaliplatin during a treatment cycle, the next Oxaliplatin injection time should be extended to 6 hours. Patients should be carefully monitored for paresthesias during the injection, and they should be advised in advance to avoid cold stimuli.

Note 1: Neurological toxicity - Neurological toxicity is classified into two types: acute paresthesias occurring within several hours or days after treatment and cumulative sensory nerve damage occurring after several treatment cycles. The incidence of hypoesthesia in the extremities and/or paresthesias after Oxaliplatin instillation is 85-90%. It may or may not be accompanied by convulsions and is caused by coldness. Symptoms resolve between two treatment cycles. The acute paresthesias syndrome of the throat has an incidence of 1-2% and occurs within several hours after administration, also caused by coldness. Symptoms are characterized by subjective dysphagia and dyspnea. However, even if objective evidence of dyspnea is not observed, symptoms resolve promptly without treatment. Oxygen may be supplied depending on symptoms.

To prevent nausea and vomiting, antiemetics (e.g., 5-HT 3 antagonists) should be administered with Dexamethasone or Methylprednisolone. Antiemetic administration can be prescribed according to the practices at the relevant site.

6.3 Dose adjustment

Dose adjustment: Based on the worst toxic response during a cycle

| Toxicity | CTC grade | Dose reduction |
| --- | --- | --- |
| Neutropenia | Grade 4(ANC<0.5/nL)persisting for ≥ 7 days | 1. First occurrence (once) 75% of Docetaxel starting dose75% of Oxaliplatin starting dose   75% of S-1 starting dose  2) Second occurrence (twice)  50% of Docetaxel starting dose  50% of Oxaliplatin starting dose  50% of S-1 starting dose  3) Third occurrence (three times)  Discontinuation of chemotherapy of this study  1) Second occurrence (twice)  75% of S-1 starting dose  2) Third occurrence (three times)  50% of S-1 starting dose  3) Fourth occurrence (four times)  Discontinuation of chemotherapy of this study |
| Febrile neutropenia | Grade 3 or Grade 4 |  |
| Thrombocytopenia | Grade 3 with bleeding  (platelet <50 k/nL), or  Grade 4 |  |
| Diarrhea, mucositis/ stomatitis and hand foot skin reaction | Grade 2 | 1) Second occurrence (twice) 75% of S-1 starting dose 2) Third occurrence (three times) 50% of S-1 starting dose 3) Fourth occurrence (four times) Discontinuation of chemotherapy of this study |
|  | Grade 3 | 1) First occurrence (once) 75% of S-1 starting dose 2) Second occurrence (twice) 50% of S-1 starting dose 3)Third occurrence (three times) Discontinuation of chemotherapy of this study |
|  | Grade 4 | 1) First occurrence (once) 50% of S-1 starting dose 2)Second occurrence (twice) Discontinuation of S-1 chemotherapy (Docetaxel and Oxaliplatin are continued) |
| Peripheral neuropathy (sensory anomaly, paresthesias) | Grade 2 | 1) First occurrence (once) 75% of Oxaliplatin starting dose 75% of Docetaxel starting dose 2) Second occurrence (twice) Discontinuation of chemotherapy of this study |
|  | Grade 3/4 | 1) First occurrence (once) Discontinuation of chemotherapy of this study |
| Other non hematological toxicity (other than nausea/vomiting and alopecia) | Grade 2 | 1) Second occurrence (twice) 75% of Docetaxel starting dose 75% of Oxaliplatin starting dose 75% of S-1 starting dose 2) Third occurrence (three times) 50% of Docetaxel starting dose 50% of Oxaliplatin starting dose 50% of S-1 starting dose 3) Fourth occurrence (four times) Discontinuation of chemotherapy of this study |
|  | Grade 3 | 1. First occurrence (once)   75% of Docetaxel starting dose  75% of Oxaliplatin starting dose  75% of capecitabine starting dose   1. Second occurrence (twice)   50% of Docetaxel starting dose  50% of Oxaliplatin starting dose  50% of capecitabine starting dose   1. Third occurrence (three times)   Discontinuation of chemotherapy of this study |
|  | Grade 4 | Discontinuation of chemotherapy of this study |

| Arms | Assigned Interventions |
| --- | --- |
| Experimental: A（DOX）  Interventions：This arm wil receive four cycles of DOX (docetaxel 60mg/m2 on day 1,oxaliplatin 130mg/m2 on day 1 and capecitabine 1,000 mg/m2 per day on days 1 to 14, repeated every 3 weeks) as neoadjuvant therapy and four cycles of Xelox (capecitabine 1,000 mg/m2 per day on days 1 to 14 and oxaliplatin 130mg/m2 on day 1, repeated every 3 weeks) as adjuvant therapy | Drug: docetaxel,0xaliplatin,capecitabine  docetaxel 60mg/m2， ivgtt，2h，d1;capecitabine 1000mg/m2 po bid d1-14; oxaliplatin 130mg/m2， ivgtt，2h，d1;q21d  Other Names:  AISU,AIHENG,AIBIN |
| Active Comparator: B（Xelox）  Interventions：This arm wil receive four cycles of Xelox (capecitabine 1,000 mg/m2 per day on days 1 to 14 and oxaliplatin 130mg/m2 on day 1, repeated every 3 weeks) as neoadjuvant therapy and four cycles of Xelox as adjuvant therapy | Drug: oxaliplatin,capecitabine  oxaliplatin 130mg/m2， ivgtt，2h，d1;capecitabine 1000mg/m2 po bid d1-14;q21d  Other Names:  AIHENG,AIBIN |
| Active Comparator: C（surgery）  Interventions：This arm wil receive eight cycles of Xelox (capecitabine 1,000 mg/m2 per day on days 1 to 14 and oxaliplatin 130mg/m2 on day 1, repeated every 3 weeks) as adjuvant therapy. | Drug: oxaliplatin,capecitabine  oxaliplatin 130mg/m2， ivgtt，2h，d1;capecitabine 1000mg/m2 po bid d1-14;q21d  Other Names:  AIHENG,AIBIN |

**7. Tumor assessment**

To follow the target lesion confirmed at Baseline, a pre-operative abdominal-pelvic CT scan will be conducted within 5 days prior to the start of Cycle 2 and after completion of Cycle 3. Additional tumor assessments can be conducted at any time if there is clinically suspected disease progression. Abdominal-pelvic CT scans at Baseline and after neo-adjuvant chemotherapy will be reviewed by a central reviewer. If tumor progression is demonstrated, the subject will complete the administration of the investigational product. If tumor progression is demonstrated during the neo-adjuvant chemotherapy period, it will be recorded in the Case Report Form, the investigational product treatment will be discontinued, and then either (1) surgery or (2) other anticancer treatment not applicable to the contents of this study will be instituted at the discretion of the investigator, among the treatments established at the relevant site. In this case, the investigator should inform the patient of tumor progression, and the subject should voluntarily determine the continuation or discontinuation of participation in this study.

Nevertheless, discontinuation of the investigational product administration does not mean discontinuation of participation in this study, and the further process will be implemented according to the follow-up plan in the protocol. In this study, discontinuation of study participation only refers to cases where the patient voluntarily withdraws consent for study participation.

**8. Surgery**

Subjects in the SC Arm will undergo surgery within 2 weeks after randomization.

Subjects in the CSC Arm will complete a total of 3 cycles of neo-adjuvant chemotherapy (3 weeks per cycle) and then undergo surgery within 1 to 3 weeks.

8.1 Pre-operative assessment (within 14 days before surgery)

The following tests should be conducted within 14 days before surgery.

• Pre-operative clinical examination: ECOG PS, physical examination, blood pressure, body weight

• Concomitant medication

• All adverse events

• Clinical laboratory safety assessment:

Hematology: Hemoglobin/Hematocrit, RBC, WBC, PMN, LYM, ANC, Platelet

Blood chemistry: Sodium, Potassium, Calcium, BUN, Creatinine, Total protein, Albumin, SGOT (AST), SGPT (ALT), Total bilirubin, Alkaline phosphatase, Glucose.

Coagulation test: PT (INR), aPTT (sec)

• 12-lead EGG

• Chest X-ray

• Other tests as clinically necessary

8.2 Surgery
The goal of the surgery is complete resection (R0). Tumor condition is explained according to
the Residual Tumor (R) Classification:
• R0; No residual cancer (negative cross-section)
• R1; Microscopically observed residual cancer (positive cross-section)
• R2; Macroscopically observed residual cancer
While the surgery method would be determined at the discretion of the investigator, it isrecommended to be based on the Surgical Manual presented in this study

8.3 Post-operative assessment
• Confirmation of the postoperative stage and the R0 resection rate: TNM pathological stage will be determined according to standardized histopathology and the AJCC 7^th^ Edition.
• In the case of a postoperative histological finding of R1 or an R2 finding in the operating room, it is considered as progressive disease. The case will be treated as the End of Treatment (EOT), and the patient will then receive standard treatment at each site and subsequently be followed for survival/death status.

However, in the case of R1, one re-surgery will be allowed for R0 resection. Depending on the result of the re-surgery, the final resection (R status) will be determined.
• Post-operative adverse events: A post-operative adverse event is determined as an event that occurs within 30 days after surgery. Adverse events that can develop as part of the surgical procedure will be reported only when considered by the investigator as an adverse event.

**9. Research procedures and data collection**

9.1 Data collection plan

The severity of adverse events will be assessed according to NCI CTCAE 3.0.

Each patient will receive a planned visit, and specific data will be recorded at different points in the visit.

All tests/tests are recommended and are subject to clinical practice.

9.2 Screening visits

The screening visit should collect medical history, including past medical history, vital signs, physical examination, ECOG PS score, blood pressure testing, electrocardiogram (ECG), blood routine (white blood cells (WBC), neutrophil (ANC)), routine urine, stool routine, gastroscopy, cardiac color Doppler ultrasound (when necessary), liver and kidney function (total bilirubin (TBIL), direct bilirubin (DBIL), ALT, AST, alkaline phosphatase (ALP), total protein (TP), albumin, urea nitrogen (BUN), creatinine (Cr), gamma-glutamyl transferase (gamma GT), lactate dehydrogenase (LDH)), electrolyte levels (potassium (K), sodium (Na), chloride (Cl), calcium (Ca), phosphorus (P)), blood coagulation function (PT, APTT), thrombin time (TT), fibrinogen (Fbg), chest radiograph (when necessary), tumor markers, and clinical indications for pregnancy testing (if applicable), etc.

9.3 Visit during treatment

Treatment period (the first ~N courses):

9.3.1 Three blood pressure tests per week for the first two weeks after administration; after that, blood pressure tests were performed twice a week throughout the entire administration period.

9.3.2 In the first cycle after the beginning of administration, routine blood tests were performed once a week, and liver and kidney function and electrolyte tests were performed once every 2 weeks. Blood routine examination every 2 weeks thereafter; blood routine, urine routine, stool routine, liver and kidney function tests, and electrolyte tests were performed every cycle.

9.3.3 After the beginning of administration, a complete set of coagulation tests (PT, APTT, TT, Fbg) and ECG should be performed once every cycle. In case of anterior cardiac pain, palpitations, and other symptoms, myocardial enzyme spectrum (creatine kinase, lactate dehydrogenase) should be immediately detected, ECG should be checked at any time, and cardiac color Doppler ultrasound should be performed. Vital signs, ECOG PS score, physical examination, and tumor marker detection should be performed every cycle.

9.3.4 One tumor efficacy evaluation was required for 2 cycles of chemotherapy and one time of preoperative after drug administration.

9.3.5 Observe and record the concomitant medication and adverse events (AE) at any time.

9.4 Follow-up visits

Patients begin the post-treatment follow-up period after the last use of the drug. Patients who experience disease progression should be followed up for survival.

The following parameters should be recorded during the follow-up period: time of disease progression or death; other tumor treatments; SAEs that occurred in the study; survival (telephone follow-up available, records to be kept).

9.5 Follow-up of adverse events

Adverse events that have not recovered at the time of drug discontinuation should be followed up, and a final evaluation should be made. All patients should be followed up for 30 days after the last dose to detect any new adverse events.

**10. Safety Evaluation**

10.1 Observation of adverse events

Adverse events (AE) are defined as any adverse medical event that occurs in a subject or clinical trial subject after receiving a drug or treatment regimen, but does not necessarily have a causal relationship with the treatment.

According to the regulations, events occurring in the pre- and post-treatment phases are also considered AE. Therefore, the reporting of safety monitoring AE or SAE should start from the time subjects enter the study and continue until the end of the study visit.

10.2 AE classification

AE was divided into grades 0-5 (NCI-CTCAE 4.0) according to the NCI "Common Acute and Subacute Toxicity Grading Standard" (see Annex 2). If an AE is not listed in the NCI toxicity grading standard, it can be determined according to the following criteria:

Ⅰ degrees (minor): feel uncomfortable, but does not affect the normal daily activities;

Ⅱ degrees (moderate): uncomfortable level is enough to reduce or affect normal daily activities;

Ⅲ degrees (serious): can't work or normal daily activities;

Ⅳ degrees (fatal): disability or death.

10.3 AE

The name, severity, occurrence time, duration, treatment measures, and outcome of AEs that occurred during the test were recorded in detail and truthfully filled in the case report form (CRF). Abnormal laboratory test data were recorded on the CRF table and repeated at least once a week until normalcy was restored or the study was completed. Adverse events occurring within 30 days of the end of the last administration are reported and documented.

10.4 Judgment of the relationship between AE and experimental drugs

The possible association between AE and experimental drugs was assessed according to the five-level classification of " Definitely relevant, probably relevant, probably irrelevant, definitely irrelevant and impossible to judge". The first three levels were judged to be related to the experimental drug. The incidence of adverse reactions was calculated using these three levels as the numerator and the number of all subjects used to evaluate safety as the denominator.

10.5 SAE

10.5.1 Definition of serious adverse events

A Serious Adverse event (SAE) refers to a medical event occurring during a clinical trial that requires hospitalization or prolonged hospitalization, is disabled, affects one's ability to work, endangers one's life or death, or causes congenital malformations. Including the following unexpected medical events:

-  lead to death of events;
-  life-threatening events (defined as the subjects of the attack, the risk of death from time to tome);
-  requiring hospitalization or extend the length of time of events;
-  can lead to permanent or severe disability/function is not complete;
-  anomaly or birth defects.
-  drug overdose.

10.5.2 pregnancy

Pregnancy during clinical trials should be reported as a serious adverse event.

10.5.3 Disease progression

Disease progression (including symptoms and signs of progression) should not be reported as a serious adverse event, but deaths due to progression during the trial or safety reporting period should be reported as a serious adverse event. Hospitalization for signs and symptoms of disease progression should not be reported as a serious adverse event. During the trial or safety reporting period, if the final outcome of cancer is death, then the event leading to death must be reported as a serious adverse event.

10.5.4 Other anti-tumor treatments

If subjects begin other antitumor therapy, for non-fatal adverse events, the reporting period is up to the commencement of new antitumor therapy. If death occurred within the reporting period for serious adverse events following the end of study treatment, it must be reported regardless of whether the patient received additional treatment.

10.5.5 Hospitalization

Adverse events resulting in hospitalization or prolonged hospitalization in clinical studies should be considered as serious adverse events. Any initial admission to a medical institution, even if it is less than 24 hours, meets this criterion.

Hospitalization does not include the following:

-  rehabilitation facility
-  a nursing home
-  routine emergency room were analyzed
-  on the day of surgery (such as outpatient/day/ambulatory surgery)
- Hospitalization or length of stay not associated with worsening of adverse events is not in itself a serious adverse event, such as:
-  due to the original disease admitted to hospital, no new adverse events occurred, nor the increase of original disease (such as: in order to check test before persistent abnormal laboratory tests so far).
-  management reasons of hospitalization (such as: annual check-up);
-  during clinical trials testing scheme provides hospital (such as: operate according to the requirement of the test plan);
-  has nothing to do with the deterioration of adverse events of elective hospital (such as: elective cosmetic surgery);
-  has scheduled therapy or surgery should be in the test plan and/or individual participants of baseline data shall be recorded;
-  only admitted to hospital because of the blood products use.

Diagnostic or therapeutic invasive procedures (e.g., surgical) and non-invasive procedures should not be reported as adverse events. However, if the disease condition that necessitated the procedure meets the definition of an adverse event, it should be reported. For instance, if acute appendicitis develops during the reporting period of an adverse event, it should be reported as an adverse event, and the appendectomy performed as a treatment for the adverse event should be documented.

10.5.6 Drug overdose

Drug overdose refers to the administration of the experimental drug to the subject within 24 hours (depending on the specific protocol) at a higher dose than prescribed by the investigator's physician. All instances of experimental drug overdose, whether related to an adverse event/serious adverse event, should be reported as a serious adverse event.

10.5.7 SAE reporting procedures

Serious adverse events should be reported from the date the subject signs the informed consent until 30 calendar days (including the 30th day) after the last use of the study drug. In the event of a serious adverse event during the trial, it must be reported to the principal investigator within 24 hours. Simultaneously, the new drug clinical research serious adverse event (SAE) report should be completed, signed, and dated. The relevant units should be immediately informed via fax, including the group leader unit, research ethics committee, the State Food and Drug Administration (CFDA), and the local food and drug administration (province or city).

Detailed documentation of serious adverse events should include information on symptoms, severity, time of occurrence, treatment duration, measures taken, follow-up duration and method, and outcome. If the investigator believes that a serious adverse event is not related to the trial drug but potentially linked to study conditions (such as termination of the original treatment or complications during the trial), the relationship should be described in detail in the Description section of the serious adverse event page of the medical record form.

**11 Statistical Analysis**

11.1 Statistical analysis of data sets

Full Analysis Set: All patients randomized to use the drug at least once will be analyzed according to the intention-to-treat (ITT) principle. For cases in which the full course of treatment was not completed, the last observation data will be carried forward to the final trial results (LOCF).

Per-protocol Set: All cases that meet the test protocol, demonstrate good compliance, have not taken prohibited drugs during the trial, and have completed the specified contents in the case report form will be included. No imputation will be performed for missing data. The efficacy of FAS and PPS will be analyzed statistically.

Safety Analysis Set: All enrolled patients who have used at least one experimental drug and have safety records after drug use will belong to the Safety Analysis Set. This dataset will be used for safety analysis.

11.2 Statistical analysis plan

All statistical analyses in this experiment will be conducted using SPSS 18 statistical analysis software. The X^2^ test will be used for counting data, t-test for measurement data, Kaplan-Meier method to calculate the survival rate, and log-rank test. Two-sided tests will be employed for all statistical tests. A p-value less than or equal to 0.05 will be considered statistically significant, and the confidence interval will be set at 95% confidence.

1. **Quality control and quality assurance**

Researchers must be clinicians trained in clinical trials and must work under the guidance of senior professionals.

The clinical ward before the test must meet the standard requirements to ensure that the rescue equipment is complete.

It is recommended that the subject be given medication by professional nursing staff to understand the medication-taking situation in detail and ensure the subject's compliance.

Filter into the group

The baseline assessment

Group A（DOX）

Group B（XELOX）

Group C（surgery）

Four cycles of DOX

Four cycles of Xelox

Surgery

Surgery

Surgery

Four cycles of Xelox

Eight cycles of Xelox
