# Supplementary figures and images for: Neoadjuvant docetaxel, oxaliplatin plus capecitabine versus oxaliplatin plus capecitabine for patients with locally advanced gastric adenocarcinoma: long-term results of a phase III randomized controlled trial
Source: Int J Surg. 2023 Sep 2;109(12):4000–8. doi: 10.1097/JS9.0000000000000692 (PMC10720837; doi:10.1097/JS9.0000000000000692)

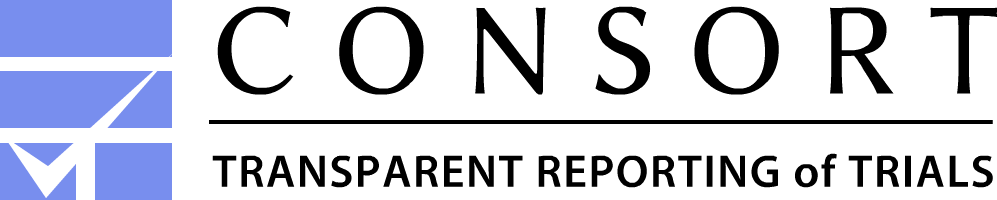


**CONST 2010 Flow Diagram**


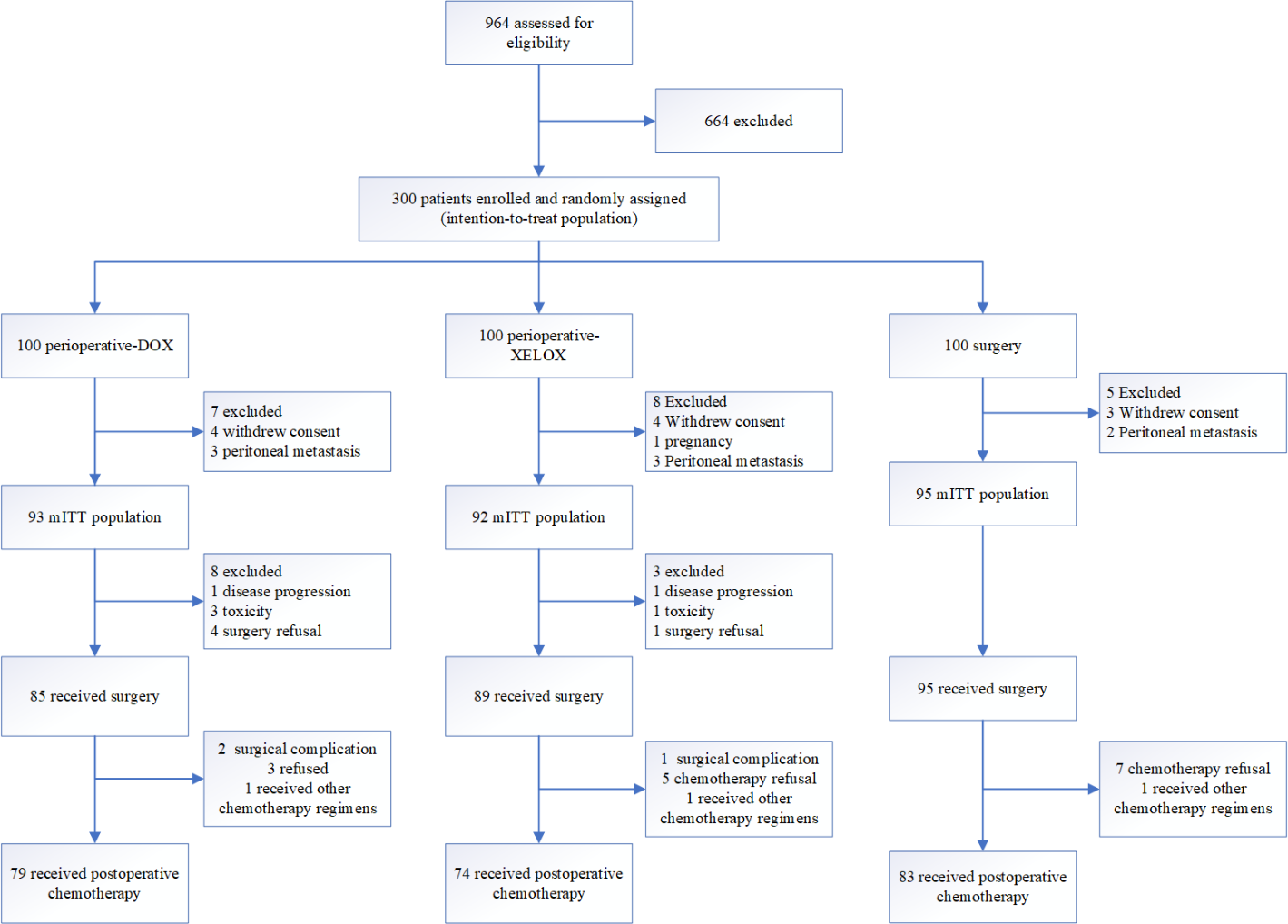

Supplement: SUPPLEMENTARY MATERIAL [file js9-109-4000-s003.docx]
