# Supplementary material for: Neoadjuvant docetaxel, oxaliplatin plus capecitabine versus oxaliplatin plus capecitabine for patients with locally advanced gastric adenocarcinoma: long-term results of a phase III randomized controlled trial
Source: Int J Surg. 2023 Sep 2;109(12):4000–8. doi: 10.1097/JS9.0000000000000692 (PMC10720837; doi:10.1097/JS9.0000000000000692)
Supplement: SUPPLEMENTARY MATERIAL [file js9-109-4000-s004.docx]

**Table 1 Serious adverse events with perioperative morbidity**

|  | DOX**(n/%)**（n=93） | XELOX**(n/%)**（n=92） | Surgery**(n/%)**（n=95） | χ^2^ | *P* value |
| --- | --- | --- | --- | --- | --- |
| Patients with at least one serious adverse event involving a perioperative morbidity | 31(33.3) | 22(23.9) | 20(21.1) | 4.008 | 0.135 |
| Surgical complication |  |  |  |  |  |
| Pneumonia | 8(8.6) | 7(7.6) | 7(7.4) | 0.11 | 0.946 |
| Pleural complication | 15(16.1) | 5(5.4) | 11(11.6) | 5.41 | 0.067 |
| Chyle leakage | 1(1.1) | 0 | 0 | 2.018 | 0.365 |
| seroperitoneum | 1(1.1) | 1(1.1.) | 1(1.1) | 0.001 | 1.000 |
| Anastomotic fistula | 1(1.1) | 1(1.1) | 0 | 1.035 | 0.596 |
| Intestinal occlusion | 0 | 0 | 1(1.1) | 1.954 | 0.376 |
