# Supplementary material for: Neoadjuvant docetaxel, oxaliplatin plus capecitabine versus oxaliplatin plus capecitabine for patients with locally advanced gastric adenocarcinoma: long-term results of a phase III randomized controlled trial
Source: Int J Surg. 2023 Sep 2;109(12):4000–8. doi: 10.1097/JS9.0000000000000692 (PMC10720837; doi:10.1097/JS9.0000000000000692)
Supplement: SUPPLEMENTARY MATERIAL [file js9-109-4000-s005.docx]

**Table 2 Adverse events in 3 groups**

|  | DOX (n=93) **(n/%)** | | | XELOX (n=92) **(n/%)** | | | Surgery (n=95) **(n/%)** | | |
| --- | --- | --- | --- | --- | --- | --- | --- | --- | --- |
|  | Grade 1-2 | Grade 3 | Grade 4 | Grade 1-2 | Grade 3 | Grade 4 | Grade 1-2 | Grade 3 | Grade 4 |
| **Gastrointestinal disorders** | | | | | | | | | |
| Nausea | 41(44.1) | 14(15.1) | 0 | 36(39.1) | 15(16.3) | 0 | 38(40) | 12(12.6) | 0 |
| Vomiting | 20(21.5) | 4(4.3) | 0 | 10(10.9) | 2(2.2) | 0 | 12(12.6) | 0 | 0 |
| Diarrhea | 34(37) | 6(6.5) | 0 | 26(28.3) | 2(2.2) | 0 | 16(16.8) | 6(6.3) | 0 |
| Constipation | 8(8.6) | 0 | 0 | 8(8.7) | 0 | 0 | 4(4.2) | 0 | 0 |
| Decreased Appetite | 46(49.5) | 8(8.6) | 0 | 38(41.3) | 8(8.7) | 0 | 40(42.1) | 6(6.3) | 0 |
| Dysphagia |  |  |  |  |  |  |  |  |  |
| **Blood and lymphatic system disorders** | | | | | | | | | |
| Anemia | 44(47.3) | 6(6.5) | 2(2.2) | 44(47.8) | 2(2.2) | 0 | 38(40) | 4(4.2) | 0 |
| Leucopenia | 54(58.1) | 16(17.2) | 4(4.3) | 50(54.3) | 17(18.5) | 4(4.3) | 46(48.4) | 15(15.8) | 0 |
| Neutropenia | 59(63.4) | 24(25.8) | 6(6.5) | 48(52.2) | 26(28.3) | 2(2.2) | 40(42.1) | 18(18.9) | 2(2.1) |
| Thrombocytopenia | 22(23.7) | 4(4.3) | 2(2.2) | 16(17.4) | 0 | 0 | 18(18.9) | 0 | 0 |
| Febrile neutropenia | NA | 2(2.2) | 0 | NA | 0 | 0 | NA | 0 | 0 |
| **General and other disorders** | | | | | | | | | |
| Neurotoxic effects | 14(15.1) | 0 | 0 | 12(13) | 0 | 0 | 8(8.4) | 0 | 0 |
| Fatigue | 46(49.5) | 10(10.8) | 0 | 38(41.3) | 7(7.6) | 0 | 34(35.8) | 8(8.4) | 0 |
| Alopecia | 58(62.4) | NA | NA | 0 | NA | NA | 0 | NA | NA |
| Weight decreased | 34(36.6) | 4(4.3) | 0 | 38(41.3) | 0 | 0 | 28(29.5) | 0 | 0 |
| Skin effects | 8(8.6) | 0 | 0 | 4(4.3) | 0 | 0 | 2(2.1) | 0 | 0 |
| **Laboratory** | | | | | | | | | |
| ALT elevation | 14(15.1) | 2(2.2) | 0 | 10(10.9) | 0 | 0 | 8(8.4) | 0 | 0 |
| GOT elevation | 10(10.8) | 2(2.2) | 0 | 12(13) | 0 | 0 | 10(10.5) | 0 | 0 |
