# Supplementary material for: Neoadjuvant docetaxel, oxaliplatin plus capecitabine versus oxaliplatin plus capecitabine for patients with locally advanced gastric adenocarcinoma: long-term results of a phase III randomized controlled trial
Source: Int J Surg. 2023 Sep 2;109(12):4000–8. doi: 10.1097/JS9.0000000000000692 (PMC10720837; doi:10.1097/JS9.0000000000000692)
Supplement: SUPPLEMENTARY MATERIAL [file js9-109-4000-s006.docx]

**Table 3 Radiological response in DOX and XELOX group according to RECIST 1.1**

| Radiological response | DOX (n=93) | XELOX (n=92) |
| --- | --- | --- |
| CR | 10(10.8) | 5(5.4) |
| PR | 31(33.3) | 24(26.1) |
| SD | 39(41.9) | 55(59.8) |
| PD | 13(14) | 8(8.7) |
