# Supplementary material for: Neoadjuvant docetaxel, oxaliplatin plus capecitabine versus oxaliplatin plus capecitabine for patients with locally advanced gastric adenocarcinoma: long-term results of a phase III randomized controlled trial
Source: Int J Surg. 2023 Sep 2;109(12):4000–8. doi: 10.1097/JS9.0000000000000692 (PMC10720837; doi:10.1097/JS9.0000000000000692)
Supplement: SUPPLEMENTARY MATERIAL [file js9-109-4000-s007.docx]

**Table 4 Radiological response in DOX and XELOX group according to the tumor volume reduction on CT**

|  | DOX (n=93) | XELOX (n=92) |
| --- | --- | --- |
| The tumor volumes before NAT (mm^3^) | 52.13 ± 25.63 | 48.34 ± 21.56 |
| The tumor volumes after NAT (mm^3^) | 42.55 ± 19.31 | 37.32 ± 28.83 |
| The effective rate* | 44.1% (14/93) | 26.1% (24/92) |

*The tumor volume reduction rate of 12.5% was measured by CT as an effective thresh old for evaluating NAT.
