# Supplementary material for: Neoadjuvant docetaxel, oxaliplatin plus capecitabine versus oxaliplatin plus capecitabine for patients with locally advanced gastric adenocarcinoma: long-term results of a phase III randomized controlled trial
Source: Int J Surg. 2023 Sep 2;109(12):4000–8. doi: 10.1097/JS9.0000000000000692 (PMC10720837; doi:10.1097/JS9.0000000000000692)
Supplement: SUPPLEMENTARY MATERIAL [file js9-109-4000-s008.docx]

**Table 5 Lymph node harvest in the three groups**

|  | DOX(n=93) | XELOX (n=92) | Surgery(n=95) | t | P |
| --- | --- | --- | --- | --- | --- |
| The  numbers of resected lymph nodes | 36.1 ± 8.4 | 35.8± 9.2 | 39.8 ± 9.7 | 1.725 | 0.104 |
| The proportion of patients with lymph node metastasis | 6.5% (218/3362) | 8% (252/3150) | 20.3% (767/3785) | 108.065 | 0. 001 |
